# Evaluation of Immunization Services for Children of Migrant Workers Along Thailand–Myanmar Border: Compliance with Global Vaccine Action Plan (2011–2020)

**DOI:** 10.3390/vaccines8010068

**Published:** 2020-02-05

**Authors:** Chamnan Pinna, Jaranit Kaewkungwal, Weerawan Hattasingh, Witaya Swaddiwudhipong, Rakdaw Methakulchart, Aree Moungsookjareoun, Saranath Lawpoolsri

**Affiliations:** 1 Tak Provincial Health Office, Muang, Tak 63000, Thailand; chamnan_pinna@yahoo.com (C.P.); rakdawmeta@yahoo.com (R.M.); 2 Department of Tropical Hygiene, Faculty of Tropical Medicine, Mahidol University, Bangkok 10400, Thailand; jaranit.kae@mahidol.ac.th; 3 Department of Tropical Pediatric, Faculty of Tropical Medicine, Mahidol University, Bangkok 10400, Thailand; weerawan.hat@mahidol.ac.th; 4 Mae Sot Hospital, Mae Sot, Tak 63110, Thailand; swaddi@hotmail.com; 5 World Health Organization Country Office for Thailand, Nonthaburi 11000, Thailand; aree@who.int

**Keywords:** immunization, migrant, Thailand–Myanmar border, global vaccine action plan, expanded program on immunization, vaccination coverage

## Abstract

Immunization is a core component of the human right to health. However, accessibility to the Expanded Program on Immunization (EPI) might be difficult among migrant children. This study aims to assess the vaccination coverage of migrant children under a mobile immunization program, initiated by the Thai government in 2014. A cross-sectional, mixed-methods study was conducted in five districts along the Thailand–Myanmar border during July–December 2018. The immunization history during their first year of life was obtained. Focus group discussions were conducted among stakeholders to explore their satisfaction toward the immunization service. Mothers/guardians of 1707 migrant children participated in the survey, with a 71% response rate. The vaccination coverage increased during 2014–2017. The highest vaccination coverage was observed for Bacillus Calmette-Guérin vaccine, with 83.2% coverage in 2017. The vaccination coverage of three doses of diphtheria-tetanus-pertussis vaccine and Hepatitis B vaccine and oral polio vaccine increased from 34.8% in 2014 to 56.3% in 2017. For measles-containing vaccine, the vaccination coverage increased from 32.4% in 2014 to 54.6% in 2017. Overall, all stakeholders were satisfied with the immunization service. Increased workload and communication barriers were the main factors that influenced the satisfaction toward the immunization program.

## 1. Introduction

Immunization is the most cost-effective health intervention and is recognized as a core component of the human right to health. In 2012, the World Health Assembly initiated the Global Vaccine Action Plan (GVAP), which aims to accelerate immunization for all people worldwide [1]. The GVAP’s mission is to expand immunization to all people by 2020, regardless of their nationality and accessibility status. In Thailand, the coverage of basic immunization, including Bacillus Calmette–Guérin vaccine (BCG), diptheria-tetanus-pertussis vaccine (DTP), oral polio vaccine (OPV), and measles-containing vaccine (MCV) has been reported to be >90% since the year 2000 [2]. In addition, the incidence of vaccine-preventable diseases (VPDs) has decreased during this period.

However, migrants are the main underserved population for immunization [3]. In the Tak province, along the Thailand–Myanmar border, the vaccination coverage among migrant children was estimated to be only 56.7% in 2013 [4]. The major barriers to immunization include continuous mobility, living in remote areas, lack of knowledge, and language barriers. In addition, illegal migrants may not want to expose themselves to public health officers because they fear getting arrested [4,5]. Inadequate vaccination coverage among migrant children could subsequently lead to epidemics of VPDs in the area among both migrant and local children [6].

In 2014, the Thailand Ministry of Public Health in collaboration with non-governmental organizations (NGOs) has put in place the Expanded Program on Immunization (EPI) to provide vaccines for migrant children along the Thailand–Myanmar border, especially in the Tak province. The Tak Provincial Health Office has worked closely with NGOs in the area, such as the Karen Department of Health and Welfare, Shoklo Malaria Research Unit, Suwannimit Foundation, and Maetao Clinic. The goal of this collaboration is to extend EPI to migrant children in the area. In remote areas where accessibility is difficult, community health volunteers were trained to administer vaccines to migrant children under their coverage areas.

Thus, this study aimed to assess the vaccination coverage among children of migrant workers after the extension of the EPI implementation from 2014 to 2017. Moreover, satisfaction regarding immunization services was assessed among different stakeholders, including mothers/guardians, vaccine providers, and local health vaccine policymakers. Findings from this study should provide insights for effective planning and EPI implementation among the hard-to-reach population.

## 2. Materials and Methods 

### 2.1. Study Design

A cross-sectional study was designed with a mixed-methods approach that was quantitative as well as qualitative. A questionnaire survey and EPI record book review were performed to determine the vaccination coverage among migrant population. Focus group discussions (FGD) were conducted among different stakeholders, including mothers/guardians, vaccine providers, and local health vaccine policymakers. The study was conducted during July–December 2018.

### 2.2. Study Areas 

**Figure 1:**
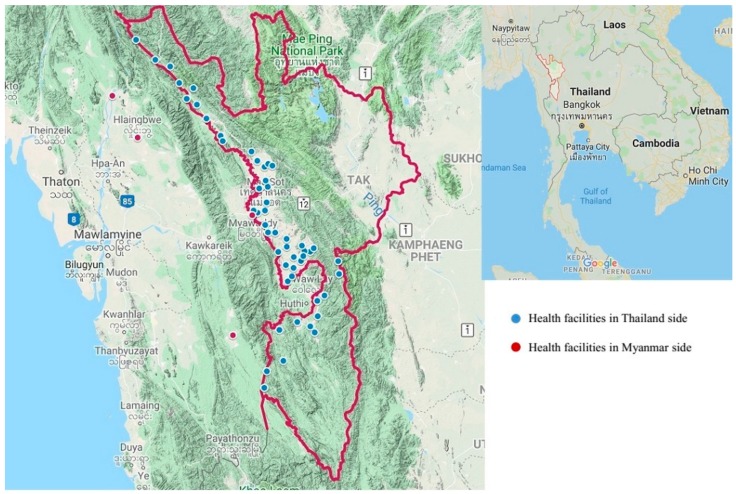
Map of health facilities providing Expanded Program on Immunization (EPI) along the Thailand–Myanmar border, Tak province, Thailand.

The study was conducted in five districts of the Tak province along the Thailand–Myanmar border: Tha Song Yang, Mae Ramat, Mae Sot, Phob Phra, and Um Phang districts. This is a mountainous area with a river that indicates the border between the two countries. The Tak province is located in the northwestern part of Thailand. It is one of the international border provinces adjacent to Kayin (Karen) State, Myanmar. For health facilities along the border, there are 47 health centers, four community hospitals, and one general hospital in the Thailand side, whereas there are four health facilities in the Myanmar side (Figure 1). All health facilities are responsible for the immunization program; vaccines under EPI are provided free-of-charge to all children regardless of their nationalities.

There are approximately 300,000 Myanmar migrants living and working in the Tak province; about 90% of these migrants are illegal migrants and do not have access to health services [7]. The epidemic of VPD, including diphtheria and measles, has been periodically reported among this population and the local people in the same area [6,8].

### 2.3. Study Population

The quantitative aspect of the study focused on migrants who were mothers or guardians of children aged 12–60 months. Migrants were defined as migrant workers from Myanmar, including both legal and illegal migrants, currently living and working along the Thailand–Myanmar border at the time of the survey. The migrants were classified according to their workplaces: industrial area, agricultural area, and remote areas (hard-to-reach areas near Eastern Myanmar where migrant children were provided EPI by mobile health worker teams). The age range of the children was selected to reflect the period of EPI for the migrant program implementation during 2014–2017. Sample size was calculated according to the World Health Organization (WHO) reference manual, 2015, which recommended random cluster sampling process for vaccination coverage studies [9]. Overall, 75 out of 172 clusters (villages) were randomly selected, with the estimated number of eligible children being 1134.

For the qualitative aspect of the study, participants were purposively selected to participate in FGDs. Participants were classified into three groups: vaccine receivers who were mothers or guardians of children aged 12–60 months, vaccine providers, and local health vaccine policymakers. Each district comprised of one vaccine receiver group (6–8 participants), one vaccine provider group (6–8 participants), and one local health vaccine policymaker group (6–8 participants).

### 2.4. Data Collection

For the quantitative study, trained data collectors (interviewers) visited every household with children in all selected clusters, asking for their participation. Informed consent was obtained from the study participants before the interview. Standard structured questionnaires were used by the interviewers. Vaccination records were obtained from the children’s immunization cards (if available) or other reliable records such as their mothers’ notes. Immunization history regarding vaccines recommended for children during their first year of life was recorded. According to the Thailand immunization schedule, children should receive one dose of BCG at birth, three doses of DTP and Hepatitis B vaccine (DTP–HepB3) given along with OPV at 2, 4, and 6 months, and one dose of MCV (MCV1) at 9 months [10]. The date of immunization of each particular vaccine was recorded to determine the completion and dropout rate. The questionnaires were checked for completeness of data before they were entered into an electronic database for further analysis.

For the qualitative study, FGDs were conducted with the three distinct groups to collect information regarding satisfaction toward the immunization service and barriers in EPI implementation. Active participants were invited to join FGDs that were held in a quiet area. Every focus group comprised of one facilitator and two note takers. A standardized set of open-ended questions was used to guide FGDs.

### 2.5. Data Analysis

Demographics of the participants in both the quantitative and qualitative studies were described. Vaccination coverage was defined as the proportion of children who received the recommended vaccines during the first year of life among all the children enrolled in the survey. According to EPI for migrant children, it was expected that the vaccination coverage for all the recommended vaccines should be above 90%. Immunization status was classified as “complete” when a migrant child received all recommended vaccines by one year of age. If a child missed at least one dose of the recommended vaccines, the child would be classified as having an incomplete immunization. Vaccination dropout rate was calculated for DTP vaccines, which required three doses during the first year of life. Dropout rate is defined as the percentage of the number of children who completed the immunization schedule from the first through the last doses divided by the cumulative total for the first dose. The WHO suggests that a dropout rate of less than 10% is considered good utilization. The percentages of vaccination coverage and dropout rates and 95% confidence intervals were calculated using the weight method adjusted for cluster data. The vaccination coverage was estimated for each vaccine and stratified by the migrants’ workplaces. Statistical analyses were performed using STATA version 12.1 (StataCorp, College Station, TX, USA).

For the qualitative analysis, conversations held during FGDs were recorded, and the information was transcribed into text. The same information; how they feel about the satisfaction of vaccination programs, barriers of vaccination program were grouped and summarized.

### 2.6. Ethics Statement

This study was approved by the Ethics Committee of the Faculty of Tropical Medicine, Mahidol University, Thailand (MUTM 2018-032-01). Information about the study was provided to the participants, and informed consent was obtained from each participant.

## 3. Results

### 3.1. Demographic Characteristics

Overall, there were 2405 eligible children in 5425 households of 75 randomly selected clusters. Among these, 1591 mothers or guardians of 1707 children participated in the quantitative study, with a 71% response rate. The general characteristics of the respondents are described in Table 1. The majority of the respondents were mothers, and more than half of the respondents were illiterate. Further, almost all (97.1%) of the respondents were from Kayin (Karen), Myanmar. About 42% of the respondents were temporary migrants, who might have crossed the border for work in Thailand during harvesting season, and 22.6% had lived in Thailand for more than 10 years.

For the qualitative study, 15 FGDs were conducted: five groups for mothers or guardians of the children, five for vaccine providers, and five for local health vaccine policymakers (Table 2). About 42.9% of mothers who participated in FGDs were illiterate, similar to the distribution observed in the quantitative survey.

### 3.2. Vaccination Coverage

**Figure 2:**
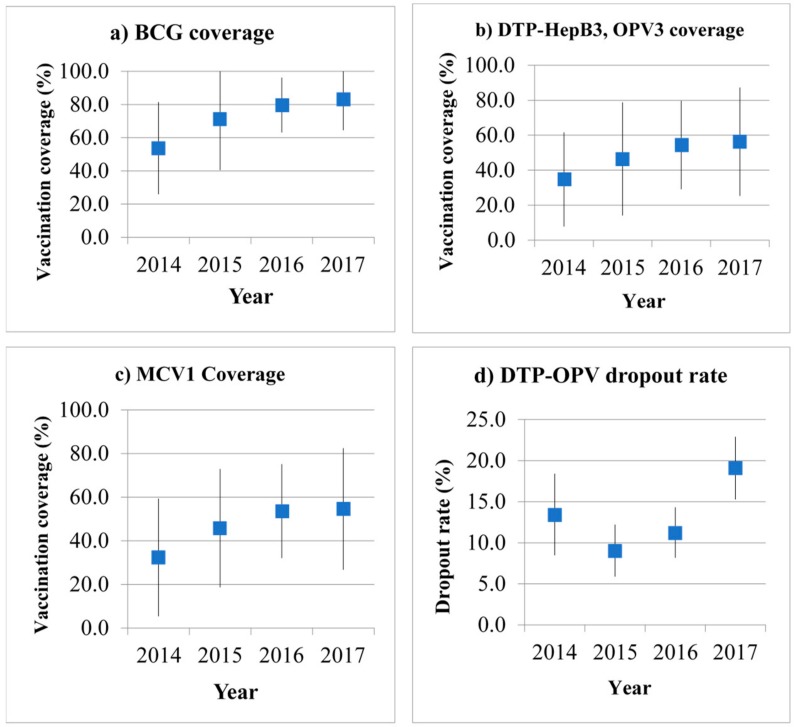
Total percentage of vaccination coverage and dropout rate during the first year of life of migrant children (2014–2017): (**a**) BCG coverage; (**b**) DTP–HepB3, OPV coverage; (**c**) MCV1 coverage; (**d**) DTP-OPV dropout rate. BCG: Bacillus Calmette-Guérin; DTP–HepB3: three doses of diptheria-tetanus-pertussis vaccine and Hepatitis B vaccine; OPV: oral polio vaccine; MCV1: one dose of measles-containing vaccine.

Overall, there was an increasing trend of vaccination coverage for all the recommended vaccines during 2014–2017 (Figure 2), although this did not reach EPI target at 90%. In addition, low vaccination coverage was more likely to be observed among children of migrants working in remote areas (Table 3).

The vaccination coverage for BCG, which is usually administered at birth, increased from 53.7% in 2014 to 83.2% in 2017. Children of migrants working in the industrial sector had higher BCG coverage than those working in agricultural and remote areas. The vaccination coverage for BCG among children of migrants working in the industrial sector was above 90% during 2015–2017 (Table 3).

The overall vaccination coverage for DTP–HepB3 and OPV3 was gradually increased from 34.8% in 2014 to 56.3% in 2017. Among the children of migrant workers in remote areas, the vaccination coverage was only 18.3% in 2014 and increased to 39.5% in 2017 (Table 3). The percentage of the vaccination coverage of MCV1 was similar to that of DTP–HepB3. The overall MCV1 coverage gradually increased from 32.4% in 2014 to 54.6% in 2017. Children of migrants in remote areas had poor vaccination coverage.

### 3.3. Dropout Rate for DTP Vaccines

The overall dropout rates for DTP–HepB3 ranged between 10.5% and 22.2%. The rate was highest in the year 2017, when about 30% of the children of migrant workers in the remote areas did not receive the three total doses of DTP–HepB. However, the dropout rate seemed acceptable among children of migrants in industrial sectors, where the dropout rate was lower than the 10% target (Table 4, Figure 2d).

### 3.4. Satisfaction of Immunization Services

#### 3.4.1. Satisfaction of Vaccine Receivers

The majority of those who received the vaccines were satisfied with the immunization program for migrant children. They appreciated that health officials came to offer the vaccines near their workplaces. Traveling to health facilities was the main challenge for them to receive vaccines for their children because most of them were illegal workers. Although the vaccination service was provided free-of-charge, sometimes the migrants cannot afford the traveling cost. In addition, traveling can increase the chances of getting caught by the Thai police. Therefore, migrant children, particularly illegal migrants, would not receive vaccination, unless there is a mobile vaccination service coming to them. Moreover, the mothers and guardians were satisfied with the service provided. The health officials always informed them about the types and numbers of vaccines that were given. Although the migrants perceived the importance of immunization, they did not remember what type of disease could be prevented by the vaccines.

#### 3.4.2. Satisfaction of Vaccine Providers

Community health volunteers who play a major role in providing mobile vaccination services to migrants were satisfied and felt proud of their responsibilities. They agreed to provide immunization to children, but they were afraid that they could not help in providing EPI for a long period of time. Because this was a voluntary work, the volunteers had to work elsewhere to earn money for their living. Therefore, there was a high turn-over rate for the health volunteers. In addition, they suggested that training courses for immunization should be conducted every year for new health volunteers and workers.

However, the Thai health workers were not satisfied with the program because it increased their workload. The Thai health workers usually provide vaccination delivery services to migrant students in the migrant learning centers. However, there is no system to monitor EPI for migrant children in those areas. Some children may get duplicate vaccination. Therefore, they suggested that there should be a supervisory authority to monitor the vaccination for migrant children. Migrant children in the learning centers should be checked for basic vaccination and have vaccination records. While providing vaccination to children of migrants working in agricultural or remote areas, it was difficult to track the next vaccination because the migrants usually move around often. Therefore, they suggested that there should be migrant health volunteers in each community who could coordinate with the health workers to follow up the next vaccination appointment. Moreover, a number of interpreters should be provided in migrant communities to assist their work. In addition, the health workers prefer to be notified if there are new migrant children arriving in the community so that they could plan their work properly. Apart from the increased workload, the Thai health workers were still willing to provide immunization services to migrant children because this could reduce the burden of VPDs not only among migrant children but also among the Thai children.

#### 3.4.3. Satisfaction of Local Health Vaccine Policymakers

The majority of local health vaccine policymakers supported the immunization program for migrant children because both the migrant and Thai populations in the area could be benefited. However, the main challenge for providing the immunization service was the lack of communication among health authorities between Myanmar and Thailand. In Eastern Myanmar, there were only few health facilities to provide immunization, especially in hard-to-reach areas where the Karen ethnic people live. People in this area usually illegally cross the border to work in Thailand and cause the outbreak of VPDs among migrants in the area. Currently, Thai healthcare workers are not responsible for providing EPI services on Myanmar soil along the border. To address this situation, a coordinating platform and structure between Thailand and Myanmar health officials, as well as related NGOs, should be established. Regular meetings should be conducted among healthcare workers in villages along the border on both sides to share and learn practical experiences together.

## 4. Discussion

Migrants are considered the main underserved population in healthcare. They usually live in poor environments and have limited access to healthcare services. This could lead to rapid transmission of communicable diseases, causing epidemics in the destination countries [7]. In Thailand, most outbreaks of VPDs, particularly diphtheria, occur among the migrant population [6,8]. Thailand has successfully provided EPI to the Thai population across the country, with vaccination coverage of 99% for BCG, DTP–HepB3, OPV3, and MCV1 [11]. However, the findings from this study show that the vaccination coverage remain low among the migrant children.

About 40% of the study population included temporary migrants who illegally crossed the border to work in Thailand for a certain period of the year. In addition, another 40% of the migrants who participated in this study had stayed in Thailand for less than 10 years, and most of them were unregistered. A similar pattern was reported among migrants along the Thailand–Cambodia border [12]. A recent survey by the Tak Provincial Health Office reported that there were about 325,000 migrants from Myanmar working in the Tak province, with only 22% of them being registered migrants. There were hundreds of cases of measles, mumps, and diphtheria reported among migrant children between 2010 and 2013 [6]. However, during 2014–2017, there was only one case of measles, one case of pertussis, and six cases of diphtheria reported among migrants. Fortunately, there were no secondary cases observed, according to the outbreak investigation report [6]. As observed by an increasing trend after EPI implementation, the implementation of the mobile immunization service to migrant children may improve the vaccination coverage; however, some challenges for the EPI implementation remained.

In this study, the vaccination coverage was highest for BCG. The coverage also reached the target at 90% among migrants whose mothers worked in the industrial sector. BCG vaccines are generally administered at birth. Therefore, children who were born at a hospital received them.

The vaccination coverage of DTP–HepB3 and OPV3 during the first year of life increased from 34.8% in 2014 to 56.3% in 2017, although the coverage was far below the national goal at 90%. In addition, the dropout rate from DTP1 to DTP3 increased from 16% in 2014 to 22.2% in 2017, leaving partially immunized children who could still be at risk for the associated diseases. The high dropout rate in 2017 was probably because of the political tension between Thailand and Myanmar health authorities that prevented the provision of vaccines to migrant children in remote areas. The coverage of MCV1 was the lowest compared with other recommended vaccines during the first year of life. Unlike BCG, MCV1 is usually administered at 9 months of age, which could be a potential challenge, especially for populations that are mobile.

Migrant children whose families work in remote areas or in the agriculture sector have lower vaccination coverage than those whose families work in the industrial sector. Migrants working in remote areas have poor access to health facilities. Most migrants in remote areas were illegal; thus, they were afraid of getting arrested if they went to the Thai government hospital, as indicated by our qualitative study. In addition, going back to Myanmar to receive vaccination was even more difficult because the health facilities in Eastern Myanmar are located far from the border. A previous study reported that after the political transition in 2010, many communities in Eastern Myanmar remained outside the reach of most official government and development programs [13,14]. The mobility of the migrant workers maybe an obstacle to the improvement of vaccination coverage among migrant children. Migrants in agricultural workplaces usually move according to the harvesting season [4,5]. Therefore, it would be difficult to track the children to administer proper vaccination.

In the mobile immunization program, community health volunteers may play a major role to facilitate the success of the program. The community health volunteers could be the contact persons who help to establish communication pathways between migrants and public health officers. In this study, about half of the migrants were illiterate and could not read the vaccination records of their children. Without a language barrier, community health volunteers should be able to find migrant children who did not receive vaccination in their community. In addition, the volunteers could provide health education and make an appointment for the next vaccination.

Moreover, vaccination cards among the migrant population should be well designed. Because these people may move around between Thailand and Myanmar, the international vaccination card with different languages (Thai, Myanmar, and Karen) would be helpful to track a child’s vaccination records. In this study, about 20% of the migrant children who were approached did not have a vaccination card because it was either lost or damaged. The vaccination card must be durable enough for migrants to carry it with them. A previous study reported that a well-designed vaccination card could be an effective intervention for increasing subsequent immunization [15]. In addition, if possible, this vaccination card could be used as a health passport when parents bring their children for vaccination at health facilities. This would reduce the pressure on the migrants in terms of being afraid of getting arrested [16].

In this study, most of the vaccination records were obtained from vaccination cards. However, about 20% of the participants did not have a vaccination card. In such cases, the immunization history was obtained from the parents’ notes or memory. Although many parents could recall the vaccines that their children had received, the vaccination date might be difficult to accurately recall. In addition, about 500 parents were excluded from the study because they did not have vaccination records and could not recall the vaccination history of their children. The overall response rate in this study was 71%. The excluded population and non-responders might have led to the underestimation of vaccination coverage in this study.

## 5. Conclusions

The vaccination coverage of the recommended vaccines during the first year of life among migrant children along the Thailand–Myanmar border remains lower than the national and global target of 90%. Population mobility and inaccessibility to health services are the main challenges faced by migrant parents. Mobile immunization program aided by community health volunteers may help to improve vaccination coverage among migrant children. However, challenges such as increased workload of local public health officers, language barriers, lack of vaccination record, and high turn-over rate of the volunteers should be considered when implementing such a program.

## Figures and Tables

**Table 1:** Demographic characteristics of respondents in the quantitative study (N = 1591).

Characteristics	Number	Percent
Respondents		
Father	103	6.5
Mother	1347	84.7
Relative	141	8.9
Education level		
Illiteracy	826	52.5
Elementary school	607	38.5
Middle and high school	137	8.7
Other	5	0.3
Ethnicity		
Myanmar	585	36.9
Karen	953	60.2
Hill tribe	10	0.6
Other	36	2.3
Length of stay in Thailand		
Temporary	646	41.9
<1 year	26	1.7
1–5 years	198	12.8
6–10 years	352	21.1
>10 years	348	22.6
Workplaces		
Industry	428	26.9
Agriculture	517	32.5
Remote area	646	40.6
Sex of their children (N = 1707)		
Male	856	50.2
Female	851	49.8

**Table 2:** Demographic characteristics of participants in focus group discussions.

Characteristics	Vaccination Relevant Respondents		
	Recipients	Providers	Policymakers
	(n = 56) (%)	(n = 40) (%)	(n = 35) (%)
Gender			
Male	0 (0.0)	16 (40.0)	11 (31.4)
Female	56 (100.0)	24 (60.0)	24 (68.6)
Ethnicity			
Myanmar	7 (12.5)	0 (0.0)	2 (5.7)
Karen	48 (85.7)	11 (27.5)	17 (48.6)
Thai	0 (0.0)	29 (72.5)	14 (40.0)
Other	1 (1.8)	0 (0.0)	2 (5.7)
Education level			
Illiteracy	24 (42.9)	0 (0.0)	0 (0.0)
Elementary school	22 (39.3)	0 (0.0)	0 (0.0)
Middle and High school	10 (17.8)	11 (27.5)	21 (60.0)
Graduate degree	0 (0.0)	28 (70.0)	6 (17.1)
Post-graduate degree	0 (0.0)	1 (2.5)	8 (22.9)

**Table 3:** Vaccination coverage during the first year of the life of children of migrants classified according to workplaces (2014–2017).

Places	Vaccination Coverage (95% CI) *			
	2014	2015	2016	2017
**BCG**				
Industry	75.0 (64.3–85.7)	93.3 (88.5–98.1)	94.0 (89.9–98.1)	98.0 (95.8–100.3)
Agriculture	73.1 (42.3–103.9)	87.9 (72.7–103.1)	82.9 (70.7–95.1)	89.5 (79.1–100.0)
Remote areas	32.6 (9.5–55.6)	50.8 (19.2–82.5)	72.3 (56.5–88.2)	68.7 (48.8–88.6)
**Total**	**53.7 (26.0–81.4)**	**71.2 (40.5–101.9)**	**79.6 (63.2–96.1)**	**83.2 (64.6–101.8)**
**DTP–HepB3, OPV3**				
Industry	60.9 (48.8–73.0)	76.2 (68.0–84.4)	77.4 (70.3–84.6)	79.7 (73.3–86.1)
Agriculture	46.0 (9.8–82.3)	60.7 (27.8–93.5)	57.4 (33.1–81.8)	59.8 (32.9–86.8)
Remote areas	18.3 (8.0–28.6)	24.9 (2.3–47.4)	44.1 (20.4–67.8)	39.5 (3.0–76.1)
**Total**	**34.8 (7.9–61.6)**	**46.4 (14.2–78.7)**	**54.4 (29.2–79.6)**	**56.3 (25.4–87.2)**
**MCV1**				
Industry	54.7 (42.3–67.0)	70.5 (61.7–79.3)	71.4 (63.7–79.2)	72.6 (65.4–79.7)
Agriculture	44.9 (8.7–81.0)	58.4 (34.9–82.0)	55.9 (34.6–77.3)	60.0 (31.8–88.2)
Remote areas	16.1 (6.7–25.5)	27.3 (5.5–49.2)	45.6 (23.0–68.3)	39.2 (5.6–72.8)
**Total**	**32.4 (5.4–59.3)**	**45.8 (18.7–72.9)**	**53.6 (32.1–75.1)**	**54.6 (26.8–82.4)**

* weighted percentage and 95% CI; Total: it is the total population (combined population from the above three categories).

**Table 4:** Dropout rate from DTP1 to DTP3 among children of migrants classified according to workplaces (2014–2017).

Places	Dropout Rate (95% CI) *			
	2014	2015	2016	2017
Industry	6.3 (−0.1–13.2)	7.1 (2.0–12.3)	8.7 (3.8–13.7)	10.5 (5.4–15.5)
Agriculture	25.3 (−23.9–74.4)	10.2 (−0.1–20.4)	17.1 (3.6–30.5)	23.8 (6.3–41.2)
Remote areas	9.0 (−7.8–25.8)	12.7 (−1.6–27.1)	11.1 (1.0–21.4)	30.1 (2.9–57.4)
Total	16.0 (−5.4–37.3)	10.5 (2.0–19.0)	12.6 (5.9–19.3)	22.2 (6.4–38.0)

* weighted percentage and 95% CI.
